# Evaluation of Mitotic Activity Index in Breast Cancer Using Whole Slide Digital Images

**DOI:** 10.1371/journal.pone.0082576

**Published:** 2013-12-30

**Authors:** Shaimaa Al-Janabi, Henk-Jan van Slooten, Mike Visser, Tjeerd van der Ploeg, Paul J. van Diest, Mehdi Jiwa

**Affiliations:** 1 Symbiant Pathology Expert Centre, Alkmaar, The Netherlands; 2 Symbiant Pathology Expert Centre, Zaandam, The Netherlands; 3 Medical Center Alkmaar, Alkmaar, The Netherlands; 4 Department of pathology, University Medical Centre Utrecht, Utrecht, The Netherlands; Innsbruck Medical University, Austria

## Abstract

**Introduction:**

Mitotic Activity Index (MAI) is an important independent prognostic factor and an integral part of the breast cancer grading system. Thus, correct estimation of this prognostically relevant feature is essential for guiding treatment decision and assessing patient prognosis.

The aim of this study was to validate the use of high resolution Whole Slide Images (WSI) in estimating MAI in breast cancer specimens.

**Methods:**

MAI was evaluated in 100 consecutive breast cancer specimens by three observers on two occasions, microscopically and on WSI with a wash out period of 4 months. MAI was also translated to mitotic scores as in grading. Inter- and intra-observer agreement between microscopic and digital MAI counts and scores was measured.

**Results:**

Almost perfect inter-observer agreements were obtained from counting MAI using a conventional microscope (intra-class correlation coefficient (ICCC) 0.879) as well as on WSI (ICCC 0.924). K coefficients reflected good inter-observer agreements among observers' microscopic mitotic scores (average kappa 0.642). Comparable results were also observed among digital mitotic scores (average kappa 0.635). There was strong to perfect intra-observer agreements between MAI counts and mitotic scores for the two diagnostic modalities (ICCC 0.716–0.863, kappa 0.506–0.617). There were no significant differences in mitotic scores using both diagnostic modalities.

**Conclusion:**

Scoring mitoses using WSI in breast cancer seems to be just as reliable and reproducible as when using a microscope. Further development of software and image quality will definitely encourage the use of WSI in routine pathology practice.

## Introduction

More than a decade ago, the practice of pathology began changing, with the introduction of slide scanners which enable the acquisition of pathology information from glass slides and translate it into a digital form commonly known as digital slides or Whole Slide Images (WSI). WSI provide the possibility of viewing and manipulating pathology samples on a computer screen in a way comparable to a conventional microscope [1]. Moreover, WSI boast many advantages over glass slides and a conventional microscope; including easy image accessibility, sharing, annotating and amenability to automated image analysis which is believed to improve the objectivity and productivity within pathology practice. These features facilitated WSI integration in different pathology applications, mainly used for education, consultation, frozen section diagnosis, quality assurance, clinico-pathological conferences and research [1]–[4]. Despite the fact that several validation studies have shown that the diagnostic performance using WSI is comparable to that of a conventional microscope [5]–[12], implementing WSI in primary diagnostics is still in its infancy. However, WSI have been used for this purpose in some pathology laboratories after carrying out their own local validation studies [13], [14]. One of the possible factors hindering WSI integration in routine pathology practice is that they have yet to be approved for primary diagnostics by the Food and Drug Administration (FDA) [15]. Additionally, the FDA has classified whole slide scanners as Class III medical devices (Slide scanner classification) necessitating extensive systematic validation studies and premarket approval before WSI can become a platform for primary diagnostics [16].

From our previous studies concerning the validation of WSI for primary diagnostics of different body systems [6]–[9], we concluded that WSI contain sufficient information for rendering most of the diagnostics within pathology. Nevertheless, we would expect that examining fine cellular details such as cellular division (mitosis) on WSI, scanned at one focal plane could pose some diagnostic difficulties. Thus, testing the validity of WSI in assessing this theoretically difficult but clinically relevant feature is crucial.

In breast cancer, tumor proliferation is one of the most important independent prognostic factors and is an integral part of the breast tumor grading system [17], [18] which has also an impact on the determination of patient treatment [19]. Different techniques may be used to estimate proliferation [20]–[22]; the most widely applicable method used in the common practice is the estimation of the mitotic activity index (MAI). MAI is defined as the numbers of mitotic figures in a given area of tumor [23]. Traditionally, MAI is scored on glass slides using light microscopy where mitosis is counted in 10 high power fields (40× magnification) or per unit area (2 mm^2^) in the most active part of the tumor [21], [23], [24]. Scoring MAI under a microscope requires the differentiation of true mitoses from similar figures such as apoptotic bodies, dark nuclei and tissue artifacts, for which a three-dimensional view and a fine microscopic focusing is required. Missing the z-axis and the ability of fine microscopic focusing on WSI scanned at one focal plane, may lead to under or overestimating MAI scores on WSI. To our knowledge, this is the first multi-observer study concerned with validating the scoring of MAI in breast cancer on the bases of WSI and digital microscope.

## Materials and Methods

This study was performed at Symbiant Pathology Expert Center in The Netherlands, consisting of pathology laboratories at three different locations serving 6 hospitals in the province of North Holland with a population of about one million people. For this study no ethics committee approval or patient consent were required. Additionally, all samples were properly coded and anonymized (Federal Guidelines).

One hundred consecutive breast cancer cases which have been previously assessed for their proliferative activity were included in this study. These concerned 6 biopsies in cases undergoing neo-adjuvant chemotherapy and 94 resections from two laboratories. From each case, one representative slide was selected by two pathologists to be used for evaluating the Mitotic Activity Index (MAI). In addition, the regions for mitosis counting were marked beforehand. This study was performed in two phases. First, MAI was scored by three observers on the same marked area on the selected glass slides using light microscopy. Thereafter, the glass slides were scanned and after a wash out period of at least 4 months WSI were presented to the same observers to recount mitosis. Table 1 details the cases included in the study.

**Table 1:** Overview of cases included for comparing mitoses counts on glass slides and whole slide images.

Diagnostic entity	Specimen type		
	Biopsies	Resections	Total
Invasive ductal carcinoma	6	76	82
Invasive lobular carcinoma		13	13
Mucinous carcinoma		1	1
Papillary carcinoma		3	3
Tubular carcinoma		1	1
Total	6	94	100

Microscopically, only cells with very evident morphology of mitosis were counted as defined before [17], [25] by absence of the nuclear membrane, clearly visible hairy extension of nuclear material (condensed chromosome), either clotted (beginning metaphase), in plane (metaphase/anaphase), or in separate clots (telophase). Doubtful cells with a hyperchromatic nucleus or cells suspected of apoptosis were excluded. The above mentioned criteria have been adopted in counting mitoses using a conventional microscope as well as WSI.

The time needed for scoring mitosis was recorded for the first ten cases in this study. Additionally, tissue quality (as poor, acceptable or good) and scan quality (as hazy, acceptable with some indistinct regions, acceptable or good) were assessed by all observers.

### MAI assessment on glass slides by conventional microscopy

Two pathologists marked the regions for mitoses counting on the H&E slides. These regions were selected at the most cellular area of the tumor, mostly located at the peripheral invasive part of the tumor as before [26]. Areas with necrosis or Ductal Carcinoma In Situ were excluded. Counting mitoses was performed at 400× magnification using a Leica light microscope equipped with 10× ocular and 40× (0.85 N/A) objective (having a field diameter of about 540 µm) in 9 consecutive fields with a total surface area of 2.06 mm^2^. The total number of mitoses in those 9 fields was taken as the MAI.

### MAI assessment on WSI

**Figure 1:**
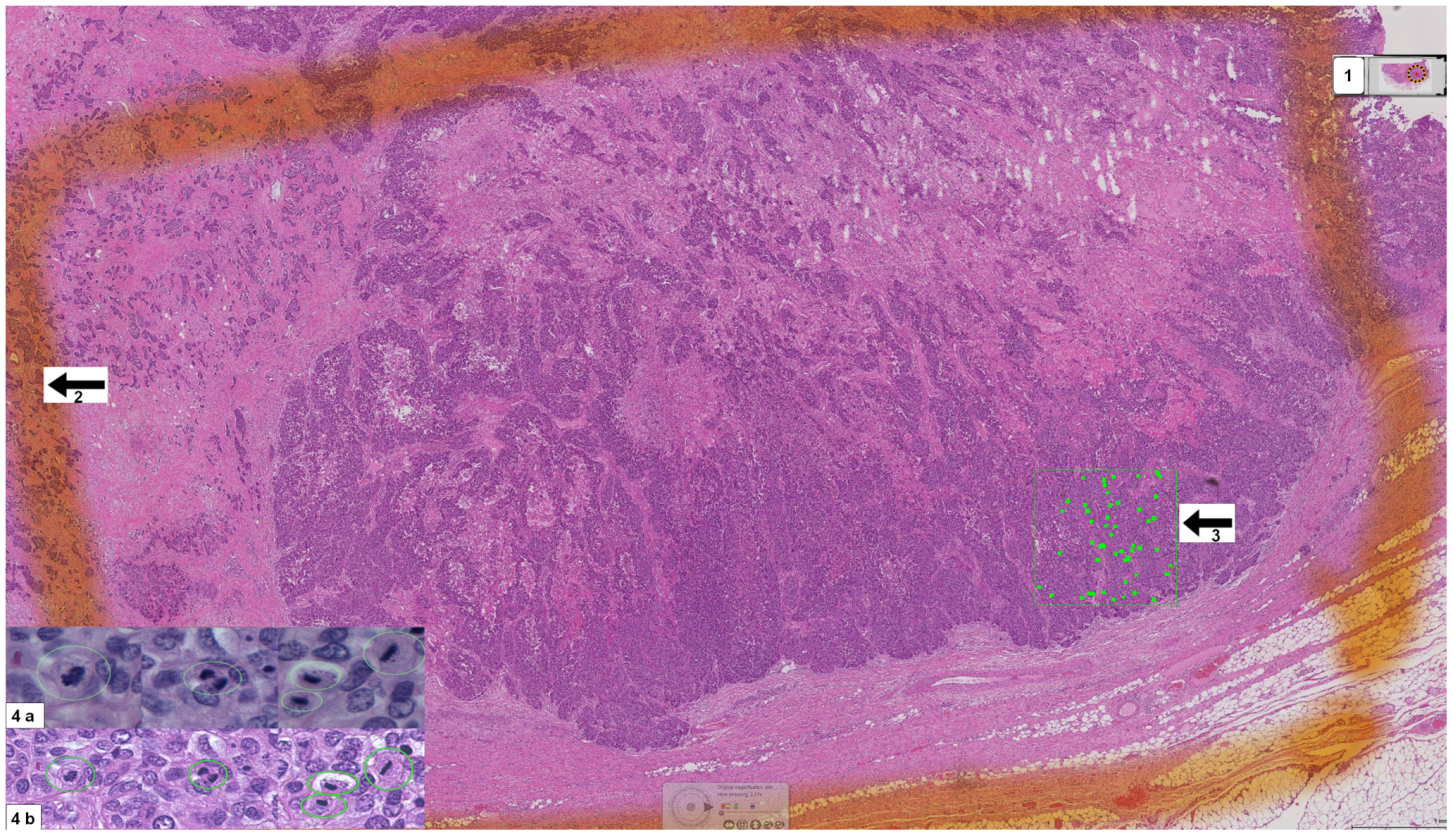
Snapshot of WSI showing the annotated areas for scoring mitosis microscopically and digitally. 1- An overview image of the WSI, 2- The defined area for counting mitosis microscopically, 3- The defined area for counting mitosis digitally, 4a- Microscopic snapshots of different mitotic figures, 4b- Digital snapshots of different mitotic figures.

Glass slides were scanned using a Leica Scanner SCN400 at 40×. The standard image viewer for Leica Scanner “Digital Image Hub” was used for annotating and exploring WSI. WSI were displayed on high resolution 30″ Barco Pathology Displays (Barco, Brussels, Belgium) having a resolution of 6 MP. Examining WSI on 40×, in an area of 2 mm^2^, about 7 screen fields fitted into the same 2 mm^2^ area annotated before on the glass slides. Each observer was asked to annotate all the mitotic figures that he could detect within this area. Afterwards mitoses annotations were counted for each observer separately. Figure 1 is a snapshot from a WSI of an invasive breast cancer showing the selected areas for counting mitosis digitally and microscopically in addition to the digitally annotated mitotic figures within a 2 mm^2^ area.

### Direct comparison of mitoses on glass slides and WSI

**Figure 2:**
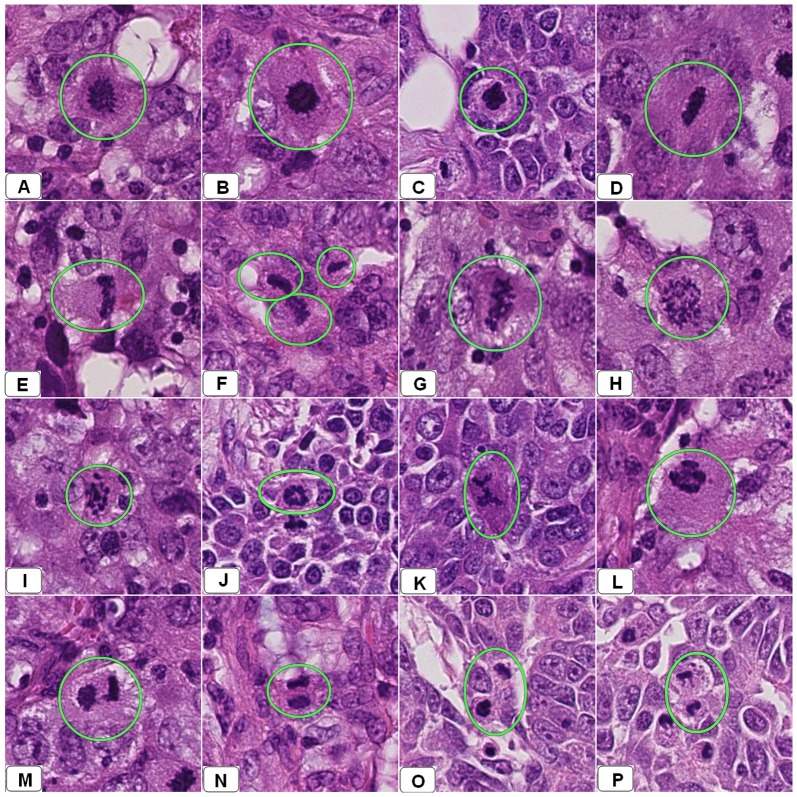
Snapshots from several WSI of several breast resections diagnosed previously as an infiltrative ductal carcinoma using a light microscopy. These snapshots showing different appearances of mitotic figures encircled by green circles. Panels A–C show cells in early metaphase. Panels D–G show different forms of mitotic division in late metaphase. Panel H–L shows different forms of anaphase. Panels M–P show cells in telophase.

**Figure 3:**
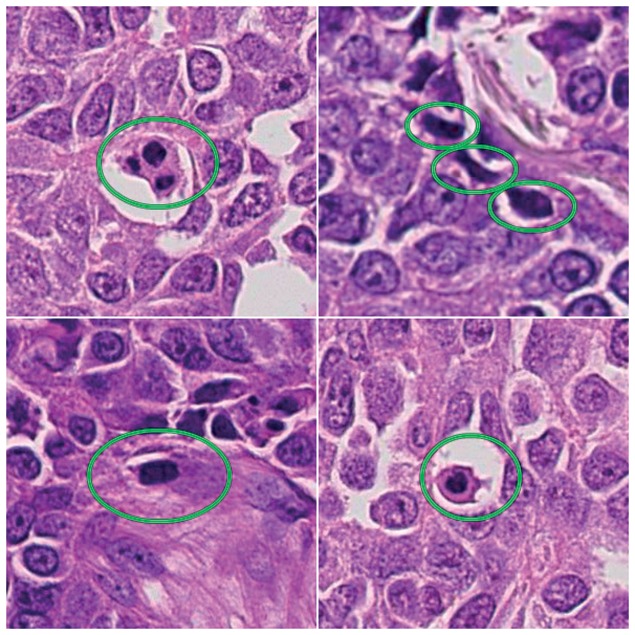
Snapshots of WSI showing the possible appearance of mitosis-like figures surrounded by green circles.

Perception of mitotic figures might be more difficult on WSI scanned at one focal plane than in conventional slides where one can perceive the 3-D structure by focusing. For this purpose and in order to gain insight into the differences in appearance using the two diagnostic modalities, mitotic figures from 15 cases were identified under a microscope and compared instantaneously with the corresponding object on WSI. Digitally, these mitoses appeared as dark nuclei with very fine projections (early metaphase), in plane with irregular margins (late metaphase), mitoses with individually dispersed chromosomes and dark ring-like shapes (anaphase) or as separated parallel dark clots (telophase). Figure 2 shows snapshots from several WSI showing these different forms of actual mitotic figures. Figure 3 shows snapshots from WSI showing different mitosis-like figures.

### Data evaluation

MAI values were transferred into mitotic scores as for grading as follows: Score 1: 0–6 mitosis/2 mm^2^, Score 2: 7–12 mitosis/2 mm^2^, Score 3: 13 mitosis or more/2 mm^2^.

Agreement was assessed between observers using the same diagnostic modality, and for each observer using the two different modalities. Intra- and inter-observers agreement for the continuous MAI was assessed using Intra Class Correlation Coefficient (ICCC), scores 0–0.2, 0.3–0.4, 0.5–0.6, 0.7–0.8, >0.8 indicating poor, fair, moderate, strong and almost perfect agreement, respectively. For mitotic scores, kappa statistics (K) were calculated to estimate inter- and intra-observer agreement [27]–[30], kappas <0.20, 0.21–0.40, 0.41–0.60, 0.61–0.80, and 0.81–1.00 indicating poor, fair, moderate, good and perfect agreement, respectively.

The level of significance was calculated using the Wilcoxon signed-rank test. Systematic differences between microscopic and digital MAI values and mitotic scores were read from the Wilcoxon signed rank test and scatter plots.

The possible effects of tissue and scan quality on differences between the conventional and digital MAI assessments were evaluated using the Mann-Whitney test.

## Results

For all observers, tissue quality did not have a significant effect on the differences between conventional and digital mitotic scores (P = 0.836, 0.187 and 0.225 for observers 1, 2, and 3, respectively).

Per observer, there was no significant effect of scan quality on the differences in scoring mitosis conventionally and on WSI (P = 0,328, 0,275 and 0.266 for observer 1, 2 and 3 respectively.

Counting mitoses on WSI was more time consuming than on glass slides. The average amount of time needed to count mitoses on glass slides ranged from 3–5 minutes versus 10–12 minutes for WSI.

### Inter-observer agreement for the same diagnostic modality

There was almost perfect inter-observer agreement among all observers in assessing MAI using a conventional microscope (ICCC 0.879) and on WSI (ICCC 0.924).

**Figure 4:**
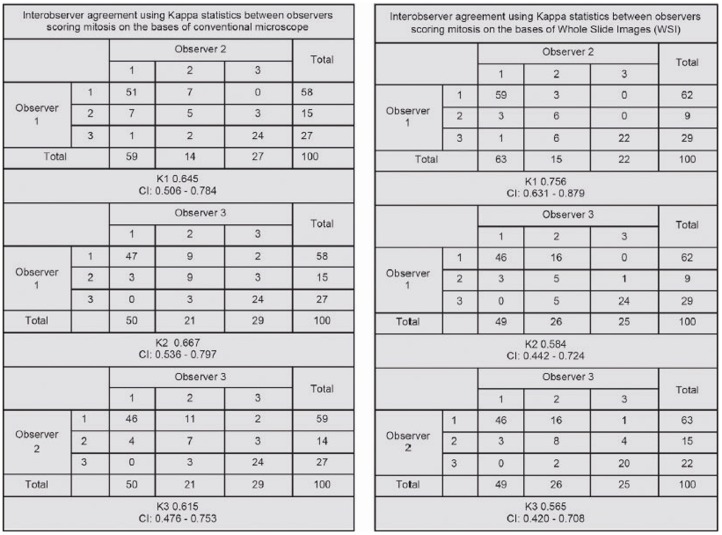
Overview of stepwise kappa statistics between observers.

Mitotic scores again yielded a good inter-observer agreements among all observers using a conventional microscope (average kappa 0.642 (K1 = 0.645, K2 = 0.667, K3 = 0.615)), and WSI (average kappa 0.635 (K1 = 0.756, K2 = 0.584, K3 = 0.565)). Figure 4 gives an overview of stepwise kappa statistics between observers.

### Intra-observer agreement for microscopic vs. WSI based mitoses counting

**Figure 5:**
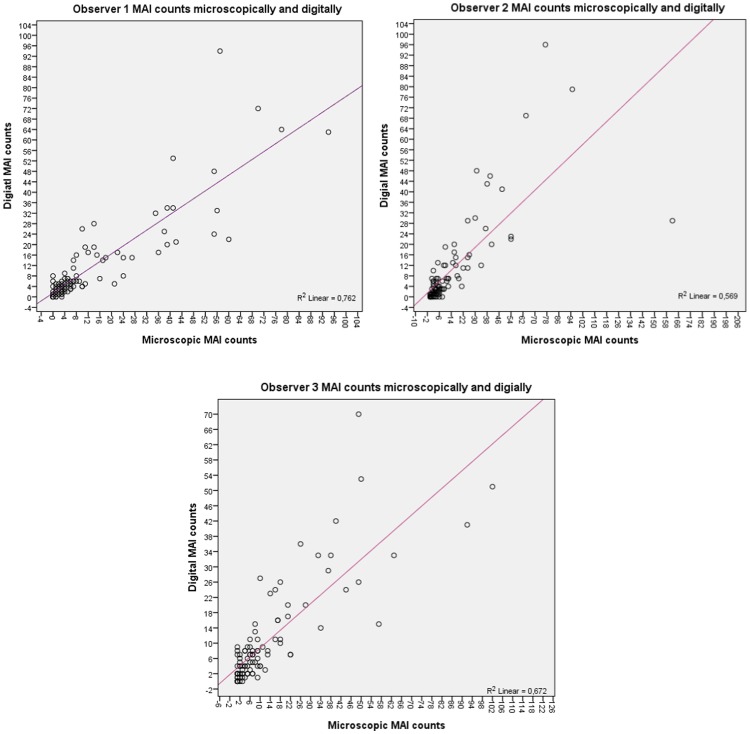
Scatter plots of microscopic versus digital MAI counts per observer indicating the tendency to underestimate of mitotic counts on WSI.

There was strong to perfect intra-observer agreement in counting mitoses when comparing both diagnostic modalities with ICCC of 0.863, 0.716, and 0.773 for observers 1, 2 and 3, respectively. In general, there was a noticeable trend towards underestimating mitotic counts on WSI if compared to microscopic mitotic counts as shown in table 2 and figure 5 per observer. Additionally, there was a better correlation between high (digital/microscopic) mitotic counts than between lower mitotic counts assessed by the two modalities. Pearson correlation coefficients between (digital and microscopic) mitotic counts from all observers sorted per mitotic scores categories are 0.455, 0.179 and 0.617 for scores 1,2 and 3 respectively.

**Table 2:** Overview of mitotic counts showing the trends towards underestimating mitotic counts when performed digitally.

		Observer 1	Observer 2	Observer 3	Total
**Mitotic counts estimation**	Digitally underestimated*	**47**	**55**	**54**	**156**
	Digitally overestimated*	**37**	**27**	**35**	**99**
	Same counts digitally and microscopically	**16**	**18**	**11**	**45**
**Total**		**100**	**100**	**100**	**300**
**Agreement of digital and microscopic mitotic counts using ICCC**		**0.863**	**0.716**	**0.773**	**0.779**

Moderate to good intra-observer agreement was observed between mitotic scores using both methods with kappa values of 0.617, 0.617, and 0.506 for observers 1, 2 and 3, respectively. Digital MAI scores were lower in 43/300 pairs of scores (microscopic and digital) and higher in 30/300 pairs (P = 0.683, 0.086 0.590 for the three observers respectively). Table 3 details per observer the intra-observer agreement of microscopic versus digital mitotic scores.

**Table 3:** Concordance rate of each observer for scoring Mitotic Activity Index (MAI) using WSI and conventional microscopy.

		Observer 1	Observer 2	Observer 3	Total
Mitotic scores estimation	Digitally underestimated*	11	15	17	43
	Digitally overestimated*	10	6	14	30
	Same scores digitally and microscopically	79	79	69	227

Agreement of digital and microscopic MAI scores	Microscopic scores	Digital scores				Microscopic scores	Digital scores				Microscopic scores	Digital scores				
		1	2	3	total		1	2	3	Total		1	2	3	Total	
	1	53	5	0	58	1	54	4	1	59	1	39	11	0	50	
	2	8	2	5	15	2	8	5	1	14	2	10	8	3	21	
	3	1	2	24	27	3	1	6	20	27	3	0	7	22	29	
	Total	62	9	29	100	Total	63	15	22	100	Total	49	26	25	100	
**Kappa**	0.617					0.617					0.506					
**Kappa CI**	0.47–0.76					0.47–0.76					0.36–0.65					
**Wilcoxon test P value**	0.683					0.086					0.590					

Per observer, digital mitotic scores have been compared with the gold standard (the conventional mitotic scores) and assessed as under- or overestimated. This assessment is general and includes all scores categories, thus scores1, 2 and 3.

## Discussion

The aim of this study was to validate the use of WSI in evaluating the MAI in breast cancer cases. MAI is an integral part of the breast cancer grading system and eventually gives an estimation of the degree of aggressiveness of the tumor and guides treatment protocols [31]. Correct evaluation of this prognostically relevant criterion is crucial since under- or overestimating mitosis scores could have important clinical implications for the patient [32].

100 breast cancer biopsies and resections were subjected to mitosis counting by three observers on two occasions; first using a conventional microscope and then after a wash-out period of at least 4 months on WSI scanned at 40×. There was almost perfect inter-observer agreement in assessing the MAI on the bases of the conventional microscope (ICCC 0.879) and on WSI (ICCC 0.924). There was also a good inter-observer agreement among three observers in scoring MAI using either a conventional microscope or WSI with average kappa values of 0.642 and 0.635, respectively. The results of this study are comparable to other studies that examined inter-observer agreement of scoring mitoses and grading of breast cancer cases by conventional microscopy only [17], [26], [33], [34].

There was a tendency to slightly underestimating the number of mitoses on WSI (table 2), but when transferring mitoses counts to mitotic scores as in grading, WSI based scores did not significantly differ from scoring mitosis using glass slides and a conventional microscope (Table 3). This indicates that scoring mitosis in breast cancer cases can be reliably done on WSI scanned at 40× magnifications and at one focal plane without influencing prognostic impact of mitotic counts.

Inter-observer agreement of the digital mitotic counts (ICCC 0.924) was slightly higher than microscopic mitotic counts (ICCC 0.879). This might be due to the fact that digital mitotic counts were performed precisely in the same annotated area of 2 mm^2^ whereas this was not the case for microscopic counting. Selection of different areas for estimating MAI and tumor heterogeneity [32] might explain the slightly lower observer agreement in counting mitosis microscopically.

Fine microscopic focusing can be helpful for differentiation of actual mitoses from mitotic-like bodies. Losing the ability of fine focusing on WSI scanned at one focal plane may theoretically impede mitosis identification. Scanning glass slides on multiple focal planes providing a z-axis to WSI may facilitate the digital evaluation of mitotic figures but increases scanning time and storage requirements which is yet impractical for routine pathology work. With the continuous improvement of scanning speed and reduction in storage cost, we expect that such limitations will be solved in the near future. Improving inter- and intra-observer reproducibility in counting mitoses can possibly be achieved by following a strict scoring protocol [17] as well as practicing more digital MAI scoring [2], [5].

Counting mitoses on WSI turned out to be more time consuming than its conventional counterpart, mainly due to cumbersome software that requires 5–6 mouse clicks to annotate one mitotic figure, and counting the total number of annotations at the end. However, annotating each mitotic figure was important for the context of this study but might not be necessary in routine practice. Adjusting the next versions of the software for research purposes to include more features such as one click annotation, an option for an automatic mitotic annotation counter and applying a 2 mm^2^ grid has been discussed with the vender. These additional features will definitely decrease scoring time and risks of error in counting mitoses and may eventually increase reproducibility. Furthermore, running automated MAI scoring on WSI would be a step forward and will assist in the objective determination of mitotic activity and hence tumor grading. Automatic detection of cancerous epithelial cells on imprint cytology slides created from breast cancer specimens [35], automated measurement of nuclear size in breast cancer [36], has already been tried with acceptable results.

The quality of WSI was generally good and adequate for use in estimating the MAI. The most frequently used level of magnification for mitosis perception was 80× digital magnification since this level of magnification offers the observer a field of view most comparable to 40× under a microscope. Also, keyboard shortcuts, which provide a more user friendly and optimal navigation within WSI, were used to move WSI in order to explore a 2 mm^2^ surface area.

Tissue quality was not optimal for every case included in this study and this might be the reason behind the discrepancies in mitotic scores. This leads to extra difficulty in counting mitosis either microscopically or digitally. Such cases were not excluded from this study as they reflect the routine mix in this pathology centre. Since poor tissue morphology can have an effect on counting mitoses [17], [32] and can rapidly compromise the quality of WSI for MAI scoring, further studies testing the effect of proper tissue morphology on digital mitosis scoring are important. However, the quality of the tissue sections included in this study did not have significant effect on the differences in scoring mitosis microscopically and digitally.

Despite of the fact that the quality of the currently produced WSI is sufficient to perform most of the diagnostics within pathology as has been approved by several validation studies, primary diagnostics based solely on WSI requires improvement of many issues such as scanning speed, image quality, software solutions and navigation interface which will definitely guarantee the successful integration of WSI routine pathology.

In conclusion, counting mitoses in breast cancer can reliably be done on high resolution WSI scanned at one focal plane. Further improvement in the software characteristics, scanning speed, and image quality will definitely encourage the use of WSI in routine practice.
